# Ecological validity of a deep learning algorithm to detect gait events from real-life walking bouts in mobility-limiting diseases

**DOI:** 10.3389/fneur.2023.1247532

**Published:** 2023-10-16

**Authors:** Robbin Romijnders, Francesca Salis, Clint Hansen, Arne Küderle, Anisoara Paraschiv-Ionescu, Andrea Cereatti, Lisa Alcock, Kamiar Aminian, Clemens Becker, Stefano Bertuletti, Tecla Bonci, Philip Brown, Ellen Buckley, Alma Cantu, Anne-Elie Carsin, Marco Caruso, Brian Caulfield, Lorenzo Chiari, Ilaria D'Ascanio, Silvia Del Din, Björn Eskofier, Sara Johansson Fernstad, Marceli Stanislaw Fröhlich, Judith Garcia Aymerich, Eran Gazit, Jeffrey M. Hausdorff, Hugo Hiden, Emily Hume, Alison Keogh, Cameron Kirk, Felix Kluge, Sarah Koch, Claudia Mazzà, Dimitrios Megaritis, Encarna Micó-Amigo, Arne Müller, Luca Palmerini, Lynn Rochester, Lars Schwickert, Kirsty Scott, Basil Sharrack, David Singleton, Abolfazl Soltani, Martin Ullrich, Beatrix Vereijken, Ioannis Vogiatzis, Alison Yarnall, Gerhard Schmidt, Walter Maetzler

**Affiliations:** ^1^Digital Signal Processing and System Theory, Electrical and Information Engineering, Faculty of Engineering, Kiel University, Kiel, Germany; ^2^Arbeitsgruppe Neurogeriatrie, Department of Neurology, Universitätsklinikum Schleswig-Holstein, Kiel, Germany; ^3^Department of Biomedical Sciences, University of Sassari, Sassari, Italy; ^4^Department of Artificial Intelligence in Biomedical Engineering, Friedrich-Alexander-Universität Erlangen-Nürnberg, Erlangen, Germany; ^5^Laboratory of Movement Analysis and Measurement, École Polytechnique Fédérale de Lausanne, Lausanne, Switzerland; ^6^Department of Electronics and Telecommunications, Polytechnic of Turin, Turin, Italy; ^7^Faculty of Medical Sciences, Newcastle University, Newcastle upon Tyne, United Kingdom; ^8^Gesellschaft für Medizinische Forschung, Robert-Bosch Foundation GmbH, Stuttgart, Germany; ^9^INSIGNEO Institute for In Silico Medicine, The University of Sheffield, Sheffield, United Kingdom; ^10^Department of Mechanical Engineering, The University of Sheffield, Sheffield, United Kingdom; ^11^Newcastle upon Tyne Hospitals NHS Foundation Trust, Newcastle upon Tyne, United Kingdom; ^12^School of Computing, Newcastle University, Newcastle upon Tyne, United Kingdom; ^13^Barcelona Institute for Global Health (ISGlobal), Barcelona, Spain; ^14^Faculty of Health and Life Sciences, Universitat Pompeu Fabra, Barcelona, Spain; ^15^CIBER Epidemiología y Salud Pública, Madrid, Spain; ^16^Insight Centre for Data Analytics, University College Dublin, Dublin, Ireland; ^17^School of Public Health, Physiotherapy and Sports Science, University College Dublin, Dublin, Ireland; ^18^Department of Electrical, Electronic and Information Engineering “Guglielmo Marconi”, University of Bologna, Bologna, Italy; ^19^Health Sciences and Technologies—Interdepartmental Center for Industrial Research (CIRISDV), University of Bologna, Bologna, Italy; ^20^Translational and Clinical Research Institute, Newcastle University, Newcastle upon Tyne, United Kingdom; ^21^Grünenthal GmbH, Aachen, Germany; ^22^Center for the Study of Movement, Cognition and Mobility, Tel Aviv Sourasky Medical Center, Tel Aviv, Israel; ^23^Department of Physical Therapy, Sackler Faculty of Medicine & Sagol School of Neuroscience, Tel Aviv University, Tel Aviv, Israel; ^24^Department of Sport, Exercise and Rehabilitation, Northumbria University, Newcastle upon Tyne, United Kingdom; ^25^Novartis Institute of Biomedical Research, Novartis Pharma AG, Basel, Switzerland; ^26^Department of Neuroscience and Sheffield NIHR Translational Neuroscience BRC, Sheffield Teaching Hospitals NHS Foundation Trust, Sheffield, United Kingdom; ^27^Digital Health Department, CSEM SA, Neuchâtel, Switzerland; ^28^Department of Neuromedicine and Movement Science, Norwegian University of Science and Technology, Trondheim, Norway

**Keywords:** deep learning (artificial intelligence), free-living, gait analysis, gait events detection, inertial measurement unit (IMU), mobility

## Abstract

**Introduction:**

The clinical assessment of mobility, and walking specifically, is still mainly based on functional tests that lack ecological validity. Thanks to inertial measurement units (IMUs), gait analysis is shifting to unsupervised monitoring in naturalistic and unconstrained settings. However, the extraction of clinically relevant gait parameters from IMU data often depends on heuristics-based algorithms that rely on empirically determined thresholds. These were mainly validated on small cohorts in supervised settings.

**Methods:**

Here, a deep learning (DL) algorithm was developed and validated for gait event detection in a heterogeneous population of different mobility-limiting disease cohorts and a cohort of healthy adults. Participants wore pressure insoles and IMUs on both feet for 2.5 h in their habitual environment. The raw accelerometer and gyroscope data from both feet were used as input to a deep convolutional neural network, while reference timings for gait events were based on the combined IMU and pressure insoles data.

**Results and discussion:**

The results showed a high-detection performance for initial contacts (ICs) (recall: 98%, precision: 96%) and final contacts (FCs) (recall: 99%, precision: 94%) and a maximum median time error of −0.02 s for ICs and 0.03 s for FCs. Subsequently derived temporal gait parameters were in good agreement with a pressure insoles-based reference with a maximum mean difference of 0.07, −0.07, and <0.01 s for stance, swing, and stride time, respectively. Thus, the DL algorithm is considered successful in detecting gait events in ecologically valid environments across different mobility-limiting diseases.

## 1. Introduction

Mobility is the ability to move about in the home and community (1). Mobility can be affected by chronic health conditions, including but not limited to neurological, respiratory, cardiac, and musculoskeletal disorders (2). Deficits in mobility have been linked with a reduced quality of life, an increased fall risk, and mortality (2, 3), therefore, mobility is regarded as an essential aspect of health (4). The most common and functionally relevant aspect of mobility that is affected by aging and chronic health conditions is walking (1, 5).

To date, the clinical assessment of mobility is based on functional tests that include short walking tasks (6–9). A common shortcoming of these functional tests is the lack of ecological validity: Walking, as measured in clinical settings, does not reflect daily life walking (3, 10–12). The transition to unsupervised monitoring of human motion in naturalistic and unconstrained daily life activities is driven mainly using wearable inertial measurement units (IMUs) (4, 13). It is noteworthy that meanwhile both European and American notified bodies for the certification of medical devices (Medical Device Regulation and Food and Drug Administration, respectively) have put focus on wearable sensors by updating their regulations for the design, pre-clinical validation, and clinical validation of devices that include wearable IMUs (13, 14). Similarly, both the European Medicines Agency and the United States Food and Drug Administration encourage the inclusion of parameters from unsupervised patient monitoring as exploratory endpoints in clinical trials (11, 15).

A critical step for the objective analysis of gait is the segmentation of gait sequences into gait cycles (16–18), i.e., the basic repetitive unit that gait is comprised of (19, 20). The beginning and end of each gait cycle, also referred to as stride, are often determined from two successive initial contacts (ICs) of the same foot (19, 20). Together with the instant at which the foot leaves the ground (i.e., final contact, FC), each stride can be divided into a stance and swing phase (18–21). ICs and FCs are commonly referred to as gait events (19, 20, 22) and are a prerequisite for any further clinical gait analysis (18). The detection of ICs and FCs from IMUs is typically done using heuristics-based algorithms (23–30). Many of these algorithms use local maxima or minima of the acceleration and/or angular velocity signals along one axis (31), which requires knowledge of the sensor-to-segment alignment (32, 33). However, in unsupervised human gait monitoring, the sensor-to-segment alignment cannot be controlled as study participants often attach the sensor themselves, for example, after showering (34). Therefore, the technical validity of these algorithms for the case of unsupervised human gait monitoring is still an ongoing challenge also due to the scarcity of labeled free-living gait data (35–37). Additionally, IMU-based gait signals are affected by disease characteristics, participant activity levels, and the exact context in which walking takes please, and therefore, any heuristics-based algorithm that was developed based on lab-based gait data might not translate directly to free-living gait (3, 11, 15, 30, 38).

In contrast to the aforementioned heuristics-based algorithms, machine learning-based algorithms do not depend on user-defined sets of rules but rather learn to recognize gait signals directly from annotated data (39–41). Hidden Markov models (HMMs), for example, were successfully applied for gait segmentation in healthy (42, 43) and pathological gait (42, 44), but only in-lab recorded gait data were used to check for validity. A recent study used HMMs to segment gait cycles from free-living gait data and reached 96% recall and 89% precision for free-living data, however, data were only from participants with Parkinson's disease (PD) (45). Although HMMs thus seem a good fit for modeling the sequential nature of the gait cycle, one still needs to define the number of discrete states beforehand, and it would be needed to have a separate model per activity if more than just gait was to be detected (46, 47). Deep learning (DL)-based algorithms provide an alternative approach that does not require any heuristic rules but rather learns relevant data representations automatically from a set of input features and reference annotations (40, 41, 48, 49). DL algorithms have been successfully applied for gait event detection from stereophotogrammetric data (50–54) and from inertial measurement unit data (34, 55), however, only for in-lab gait data.

Therefore, the specific aim of the current study was to determine whether a previously in-lab validated DL-based algorithm (34) for the detection of ICs and FCs can be used for the detection of gait events in pre-extracted real-life walking bouts in a heterogeneous cohort of different mobility-limiting diseases. For the current study, walking bouts were defined according to the recently published consensus framework for digital mobility monitoring (2).

## 2. Materials and methods

### 2.1. Data collection

#### 2.1.1. Study participants

As part of the Mobilise-D technical validation study (56), a convenience sample of 108 participants was recruited at five independent study sites (Newcastle upon Tyne Hospitals NHS Foundation Trust, UK, Sheffield Teaching Hospitals NHS Foundation Trust, UK, Tel Aviv Sourasky Medical Center, Israel, Robert Bosch Foundation for Medical Research, Germany, University of Kiel, Germany). The sample represented five mobility-limiting disease cohorts [congestive heart failure (CHF), chronic obstructive pulmonary disease (COPD), multiple sclerosis (MS), Parkinson's disease (PD), and proximal femoral fracture (PFF)] and a cohort of healthy older adults (HA) (56). These cohorts cover a range of walking speed, mobility challenges, and potential events that are of clinical interest, such as improving vs. worsening of function, falls, hospitalization, nursing home admission, and death. Furthermore, as the participants were recruited at five different sites across Europe, they ensured a geographical representation and covered a diverse representation of healthcare organization, such as in- vs. outpatient care, as well as public vs. private health services (1, 56). Participants needed to be able to walk 4 m independently, to give informed consent, and have a Montreal Cognitive Assessment score > 15 (57). A detailed description of inclusion and exclusion criteria is provided elsewhere (56), and ranges of values for cohort-specific clinical scales are detailed in Table 1.

**Table 1:** Dataset details for training, validation, and testing sets, including the total number of bouts and strides.

**Set**	**Cohort**	**Number of participants**	**Age (years)**	**Height (cm)**	**Weight (kg)**	**Clinical scale (mean [min, max])**	**Number of bouts**	**Number of strides**
Training	CHF	8	69 (13)	177 (8)	86 (20)	KCCQ: 81.8 [37.0, 96.3]	189	11326
	COPD	11	70 (9)	169 (6)	73 (14)	CAT: 21.1 [6.0, 33.0] FEV_1_: 1.7 [0.9, 2.7]	187	6562
	MS	12	47 (8)	171 (14)	80 (23)	EDSS: 3.5 [1.0, 6.5]	139	6216
	PD	12	70 (7)	175 (6)	79 (16)	HandY: 2.0 [1.0, 3.0] UPDRS: 31.8 [6.0, 54.0]	165	7574
	PFF	10	83 (6)	172 (9)	71 (16)	SPPB: 7.3 [0.0, 12.0]	151	5838
	HA	12	71 (7)	168 (10)	76 (11)		245	13597
Validation	CHF	2	74 (13)	172 (21)	87 (3)	KCCQ: 94.8 [89.6, 100.0]	41	1210
	COPD	3	69 (14)	171 (10)	69 (12)	CAT: 15.3 [6.0, 26.0] FEV_1_: 1.4 [1.3, 1.6]	68	1890
	MS	3	42 (15)	172 (13)	97 (24)	EDSS: 2.5 [1.5, 4.0]	24	863
	PD	3	70 (7)	174 (6)	79 (21)	HandY: 2.3 [2.0, 3.0] UPDRS: 28.0 [24.0, 33.0]	61	3466
	PFF	2	71 (1)	164 (8)	60 (9)	SPPB: 5.0 [1.0, 9.0]	31	1087
	HA	4	72 (4)	163 (10)	77 (18)		126	4952
Testing	CHF	2	65 (13)	168 (1)	77 (16)	KCCQ: 66.7 [47.9, 85.4]	10	407
	COPD	3	69 (8)	166 (3)	80 (18)	CAT: 18.7 [13.0, 24.0] FEV_1_: 1.4 [0.8, 2.3]	79	2346
	MS	3	58 (12)	172 (16)	87 (25)	EDSS: 4.7 [3.0, 6.0]	53	2576
	PD	3	70 (11)	166 (11)	73 (8)	HandY: 2.3 [2.0, 3.0] UPDRS: 24.3 [7.0, 41.0]	38	2448
	PFF	2	76 (6)	168 (8)	75 (28)	SPPB: 6.5 [3.0, 10.0]	21	1649
	HA	4	73 (3)	164 (11)	72 (10)		94	3674

CAT, COPD assessment test; CHF, congestive heart failure; COPD, chronic obstructive pulmonary disease; EDSS, Expanded disability status scale; FEV_1_, Forced expiratory volume in 1 s; HA, healthy adults; HandY, Hoehn and Yahr scale; KCCQ, Kansas City cardiomyopathy questionnaire; MS, multiple sclerosis; PD, Parkinson's disease; PFF, proximal femoral fracture; SPPB, short physical performance battery; UPDRS, Movement Disorder Society-sponsored Unified Parkinson's Disease Rating Scale, part III. Age, height, and weight are presented as mean (standard deviation), and the clinical scales are presented as mean [minimum, maximum].

#### 2.1.2. Study protocol

Study participants were equipped with the INertial module with Distance sensors and Pressure insoles (INDIP) system that included both pressure insoles (PIs) and IMUs to record movement signals from both feet and the lower back (27, 58, 59). Participants wore the INDIP system for 2.5 h in their habitual environment, e.g., home, work, community, and/or outdoor environment, which was chosen by the participant, with no specific restrictions (56). To capture the largest possible range of activities, participants were provided with a list of activities that could be included if relevant to their chosen environment (e.g., rising from a chair, walking to another room, and walking outdoors). No supervision or structure as to how these tasks were completed was given to the participants. The duration of the observation has been established as a trade-off between experimental, clinical, and technical requirements (56).

### 2.2. Data processing

#### 2.2.1. Data preparation

Data from the INDIP system were synchronized by setting the clock to have the same timestamp for all the sensors between the left and right foot, and values were recorded at a sampling frequency, *f*_*s*_, of 100 Hz. As input to the DL algorithm, only the raw accelerometer and gyroscope data from both feet were used. Data were split into three different datasets: a training set, a validation set, and a testing set (40, 41). For this purpose, for each of the six cohorts, data from approximately 20% of the participants were assigned to the testing set, data from another 20% of the participants were assigned to the validation set, and data from the remaining participants were used as the training set.

The validation set was used to find an optimal network architecture using grid search (60), and the training set was used to optimize the corresponding model parameters (40, 41). The testing set was only used for the final evaluation, and notably, the numbers presented in the Section Results only corresponded to the performance of the testing set.

#### 2.2.2. Reference system

For all data, the gait events, that is both ICs and FCs, were detected separately from the PIs and IMUs from the INDIP system that is described in detail elsewhere (61) to meet the emerging demands associated with reproducibility and replicability in biomedical research and regulatory qualification (62). Then, the results were combined, and priority was given to the PIs in case both modalities detected an event (63). For the PIs, foot-ground contact was defined when at least three sensing elements from the PI belonging to the same spatial neighborhood were consecutively activated and deactivated (64). For the IMUs, an existing algorithm, originally designed for shank-worn IMUs, was adapted for use with foot-worn IMUs. Previously, it was validated for the detection of supervised gait events in older, hemiparetic, parkinsonian, and choreic gait (27, 65) and across multiple research centers for parkinsonian and mildly cognitive impaired gait (66).

From these gait events, walking bouts (WBs) were formed by merging information from left and right strides (27, 28). Each WB represented a gait sequence with a minimum of two left and two right strides (2, 63). Here, strides were only considered valid if (i) the stride duration was between 0.2 and 3 s and (ii) the stride length was minimally 0.15 m. A resting period of 3 s determined consecutive WBs, thus, each WB could contain a resting period of ≤3 s.

For the current study, we analyzed only those WBs that lasted ≥10 s (67–70) and for which both the INDIP's PIs and IMUs were used for determining the gait events. These gait events were considered as reference annotations for training and evaluating the DL algorithm.

#### 2.2.3. Deep learning algorithm

**Figure 1:**
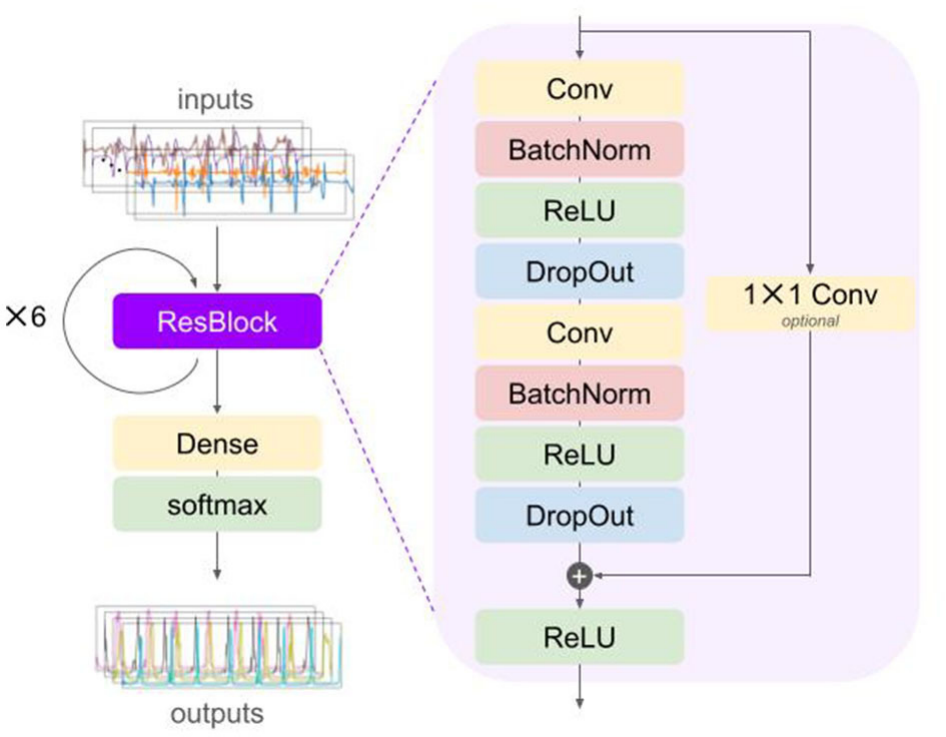
Schematic depiction of the deep learning model architecture with a residual block (ResBlock) that is repeated (in this case, six times) before a dense and softmax layer are applied. Inputs to the network are the raw accelerometer and gyroscope data of both left and right inertial measurement units. The outputs are estimated probabilities for each of the gait events for each time step. BatchNorm, batch normalization; Conv, convolution; DropOut, dropout; ReLU, recitified linear unit.

The DL algorithm was based on the neural network (NN) that was previously validated on in-lab gait data from shank-worn IMUs worn by participants with different neurological diseases (34, 71). At the core of the NN was a temporal convolutional network (TCN) (72, 73). The TCN was built from stacking residual blocks (74) with an exponentially increasing dilation factor for the convolutional layers (Figure 1).

Specifically, each residual block comprised two sequences of a dilated convolution (Conv) layer (75), a batch normalization (BatchNorm) layer (76), a rectified linear unit (ReLU) activation layer, and a dropout layer (77). A residual connection was used to perform convolution with a kernel size of 1 in case the number of feature maps did not match the number of input channels (72, 73). The outputs of the second dropout layer and the residual connection were summed elementwise and inputted to a ReLU activation layer. The convolution layers consisted of 64 filters with a kernel size of 3 and a dilation factor of 2^*m*−1^ with *m* = 1, ⋯ , *N*_*dil*_ for the *m*-th residual block (with *N*_*dil*_ = 6, the number of residual blocks, and thus, the maximum dilation factor was 2^5^ = 32).

The outputs of the last residual block were passed through a fully connected (also referred to as dense) layer followed by a softmax activation layer (78, 79). The final outputs were then regarded as probability that a certain gait event took place at the given time step, *t*_*n*_.

### 2.3. Evaluation

As in our previous studies (34), the performance was evaluated with the testing set only. The trained model was used to predict the probability that any gait event occurred from the IMU data. Peak probabilities, with a minimum probability, Δ_Pr_ = 0.5, and a minimum interpeak distance, Δ_*t*_ = 0.5 s, were considered detected events.

Performance was evaluated for the overall detection performance, time agreement between predicted and annotated gait event timings, and time agreement between subsequently derived stride-specific gait parameters.

#### 2.3.1. Overall detection performance

The overall detection performance quantified how many of the annotated gait events were detected (true positives), how many of the annotated gait events were not detected (false negatives), and how many of the detected events were not annotated (false positives). From these numbers, the recall (also referred to as sensitivity) and precision (also referred to as positive predictive value) were calculated as follows:

(1) $$\begin{array}{l} recall=\frac{\#\;true\;positives}{\#\;true\;positives+\#\;false\;negatives}, \end{array}$$

(2) $$\begin{array}{l} precision=\frac{\#\;true\;positives}{\#\;true\;positives+\#\;false\;positives}. \end{array}$$

Thus, the recall represented the fraction of annotated events that were detected, and the precision represented the fraction of events that were truly gait events.

Here, in case the absolute time difference between an annotated and predicted event was ≤250 ms, it was considered a true positive event (30, 34, 80, 81) (in other words, a tolerance window of 500 ms centered around the reference timing was used).

#### 2.3.2. Time agreement

For all correctly detected gait events (true positives), the time agreement between the detected and annotated event timings was quantified by

(3) $$\begin{array}{l} \epsilon =t_{ref}-t_{pred}, \end{array}$$

where *t*_*pred*_ is the timing corresponding to the peak probability and *t*_*ref*_ is the timing of the INDIP-derived annotations.

As a robust measure for the time agreement and its spread, the median time error and the inter-quartile range (IQR) were computed (82), and time agreements were visualized using box plots.

#### 2.3.3. Stride-specific gait parameters

For those strides where both ICs and the FC in between were detected, the stance, swing, and stride times were computed (19, 20, 83). Stance time was the time between an FC and the preceding IC of the same foot, swing time was the time between an IC and the preceding FC of the same foot, and stride time was the time between two consecutive ICs of the same foot (34, 83).

For each of these temporal gait parameters, the mean time difference and the limits of agreement (LoA) based on a 95% confidence interval (CI) were computed (82). Differences were visualized using Bland–Altman plots (84, 85).

## 3. Results

### 3.1. Demographics

Data were collected from 108 different participants, and eventually data from 99 participants were used for the current study (Table 1). Data from the other participants were excluded due to incomplete or missing data from the INDIP system or because no WBs ≥ 10 s were recorded. Eventually, the DL-based algorithm was evaluated for its performance in detecting gait events of 13,100 strides divided over 295 bouts recorded from 17 participants in the testing set.

### 3.2. Overall detection performance

The overall detection performance was quantified by the number of true positives, number of false negatives, and number of false positives. From these numbers, the recall and precision were calculated (Table 2). In total, from 13,134 ICs, the algorithm detected 12,985 events (i.e., 99%) and missed 169 events (i.e., 1%), and similarly, from 12,838 FCs, the algorithm detected 12,747 events (i.e., 99%) and missed 91 events (i.e., 1%). When evaluated per cohort, the recall for the IC detection was ≥98%, and the precision was ≥96%. Similarly, the recall was ≥99%, and the precision was ≥94% for FC detection for all cohorts.

**Table 2:** Overall detection performance of initial and final contacts evaluated per cohort.

**Cohort**	**Initial contacts**						**Final contacts**					
	**TP**	**FN**	**FP**	**R (%)**	**P (%)**	**F1 (%)**	**TP**	**FN**	**FP**	**R (%)**	**P (%)**	**F1 (%)**
CHF	408	3	18	99	96	97	401	1	23	100	95	97
COPD	2,294	58	86	98	96	97	2,235	37	147	99	94	96
MS	2,563	19	72	99	97	98	2,518	12	95	100	96	98
PD	2,431	23	40	99	98	99	2,411	3	55	100	98	99
PFF	1,642	11	15	99	99	99	1,614	18	45	99	97	98
HA	3,627	55	98	99	97	98	3,568	20	141	99	96	98

CHF, congestive heart failure; COPD, chronic obstructive pulmonary disease; F1, F1 score; FN, false negative; FP, false positive; HA, healthy adults; MS, multiple sclerosis; P, precision; PD, Parkinson's disease; PFF, proximal femoral fracture; R, recall; TP, true positive.

### 3.3. Time agreement

**Figure 2:**
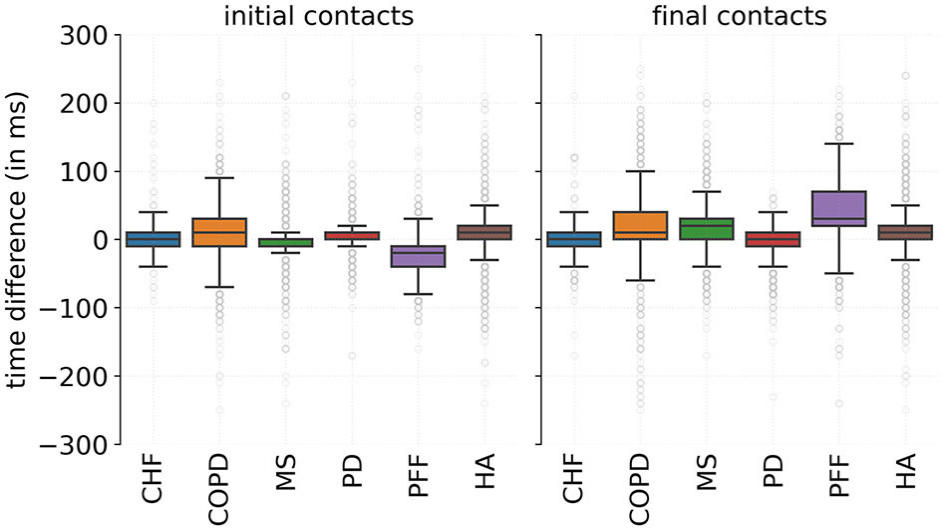
Time difference between the predicted and reference events timings for initial and final contacts evaluated per cohort. A positive time difference corresponded to an advanced detection. CHF, congestive heart failure; COPD, chronic obstructive pulmonary disease; MS, multiple sclerosis; HA, healthy adults; PD, Parkinson's disease; PFF, proximal femoral fracture.

For all the correctly detected events, i.e., true positives, the difference between the detected event timing and the annotated event timings was calculated according to Equation (6). The median time error was close to 0 s with the IQR enclosing a zero difference for both ICs and FCs for all cohorts, except for the PFF cohort (Figure 2). The PFF cohort showed a median time error of −0.02 s and an IQR of 0.03 s for IC detection, and a median time error of 0.03 s and IQR of 0.05 s for FC detection (Table 3).

**Table 3:** Time differences between the predicted event timings and the annotated event timings evaluated per cohort.

**Cohort**	**Initial contacts**		**Final contacts**	
	**Median (ms)**	**IQR (ms)**	**Median (ms)**	**IQR (ms)**
CHF	0	20	0	20
COPD	10	40	10	40
MS	0	10	20	30
PD	10	10	20	30
PFF	−20	30	30	50
HA	10	20	10	20

CHF, congestive heart failure; COPD, chronic obstructive pulmonary disease; MS, multiple sclerosis; HA, healthy adults; IQR, inter-quartile range; PD, Parkinson's disease; PFF, proximal femoral fracture.

### 3.4. Stride-specific gait parameters

**Figure 3:**
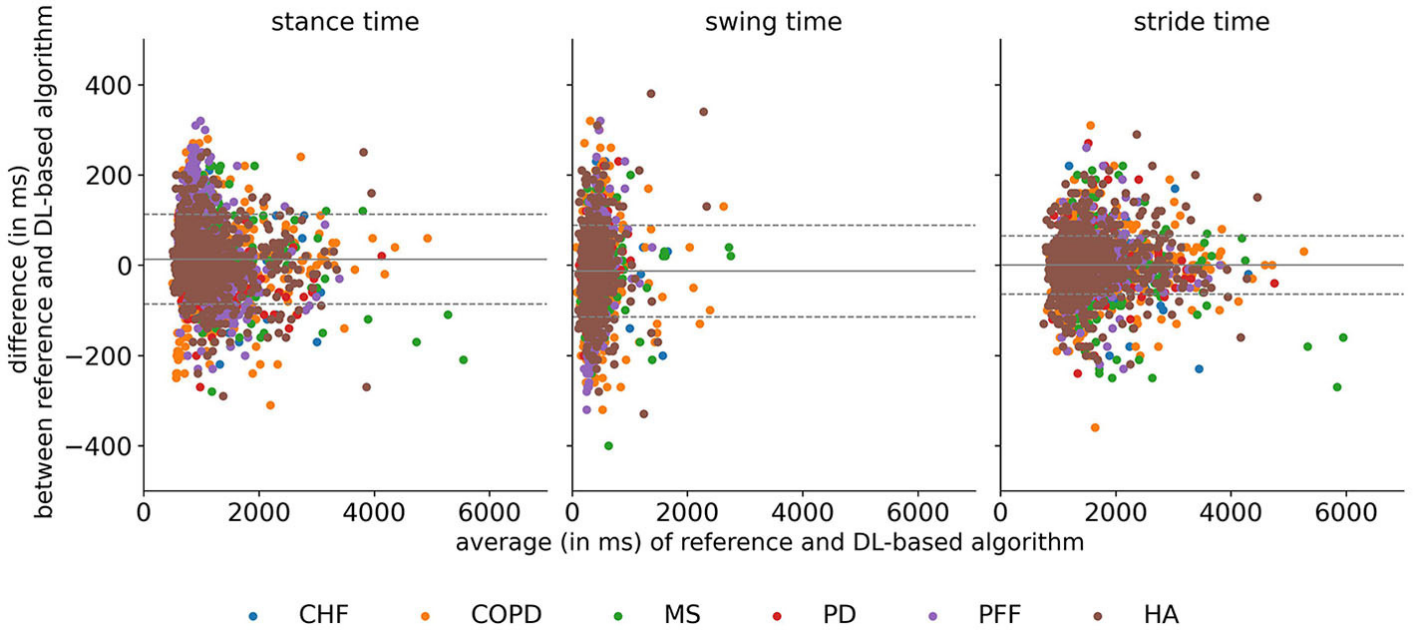
Bland–Altman plots for the stance, swing, and stride times evaluated per cohort. The gray solid line corresponds to the overall mean difference, and the dashed lines correspond to the mean difference ± 1 standard deviation. CHF, congestive heart failure; COPD, chronic obstructive pulmonary disease; DL, deep learning; HA, healthy adults; MS, multiple sclerosis; PD, Parkinson's disease; PFF, proximal femoral fracture.

For those strides that had two correctly detected ICs and a correctly detected FC in between, stride-specific temporal gait parameters (i.e., stance time, swing time, and stride time) were calculated. For all cohorts, the mean differences between the stance, swing, and stride times derived from the detected events and those derived from the annotations were close to zero with the LoA encapsulating a zero-mean difference (Figure 3). Notably, for the PFF cohort, the mean time difference for the stance time was +0.07 s, and the mean time difference for the swing time was −0.07 s, which resulted in a zero-mean difference for the stride time (Table 4). Similarly, for all gait phases, the absolute errors were 0.04 s or less for all cohorts, except the PFF cohort (Table 5). This resulted in a relative time error for the stride times of ≤2% across all cohorts, but for the swing times, the relative time error for the PFF cohort was 27%, and for the COPD cohort, it was 12%.

**Table 4:** Mean differences (bias) and limits of agreement for a 95% confidence interval for the stance, swing, and strides evaluated for each cohort.

**Cohort**	**Stance time**		**Swing time**		**Stride time**	
	**Mean difference (s)**	**LoA (s, s)**	**Mean difference (s)**	**LoA (s, s)**	**Mean difference (s)**	**LoA (s, s)**
CHF	−0.00	(−0.08, 0.07)	0.00	(−0.07, 0.07)	−0.00	(−0.07, 0.07)
COPD	0.01	(−0.11, 0.13)	−0.01	(−0.13, 0.11)	0.00	(−0.08, 0.08)
MS	0.02	(−0.05, 0.10)	−0.02	(−0.10, 0.05)	−0.00	(−0.06, 0.06)
PD	−0.01	(−0.06, 0.04)	0.01	(−0.04, 0.06)	0.00	(−0.05, 0.05)
PFF	0.07	(−0.06, 0.19)	−0.07	(−0.20, 0.07)	0.00	(−0.07, 0.07)
HA	0.00	(−0.07, 0.08)	−0.00	(−0.09, 0.08)	0.00	(−0.07, 0.07)

CHF, congestive heart failure; COPD, chronic obstructive pulmonary disease; HA, healthy adults; LoA, limits of agreement; MS, multiple sclerosis; PD, Parkinson's disease; PFF, proximal femoral fracture.

**Table 5:** Stance, swing, and stride times obtained from the reference and the DL algorithm, and the absolute and relative time errors for comparison.

**Gait phase**	**Cohort**	**Reference system**		**DL algorithm**		**Absolute error**		**Relative error**	
		**s**	**(s, s)**	**s**	**(s, s)**	**S**	**(s, s)**	**%**	**(%, %)**
Stance	CHF	0.93	(0.90, 0.97)	0.94	(0.91, 0.97)	0.03	(0.02, 0.03)	3	(2, 3)
	COPD	0.93	(0.91, 0.94)	0.92	(0.90, 0.93)	0.04	(0.04, 0.05)	5	(5, 5)
	MS	0.98	(0.97, 0.99)	0.96	(0.94, 0.97)	0.03	(0.03, 0.03)	3	(3, 3)
	PD	0.80	(0.79, 0.80)	0.81	(0.80, 0.81)	0.02	(0.02, 0.02)	2	(2, 2)
	PFF	0.90	(0.88, 0.91)	0.83	(0.82, 0.84)	0.08	(0.08, 0.08)	9	(9, 9)
	HA	0.84	(0.83, 0.85)	0.84	(0.83, 0.85)	0.03	(0.02, 0.03)	3	(3, 3)
Swing	CHF	0.41	(0.40, 0.42)	0.41	(0.39, 0.42)	0.02	(0.02, 0.03)	6	(5, 7)
	COPD	0.43	(0.42, 0.43)	0.43	(0.43, 0.44)	0.04	(0.04, 0.05)	12	(11, 13)
	MS	0.41	(0.41, 0.42)	0.44	(0.43, 0.44)	0.03	(0.03, 0.03)	9	(8, 9)
	PD	0.41	(0.40, 0.41)	0.40	(0.39, 0.40)	0.02	(0.02, 0.02)	4	(4, 4)
	PFF	0.34	(0.34, 0.35)	0.41	(0.40, 0.41)	0.08	(0.08, 0.08)	27	(26, 28)
	HA	0.36	(0.36, 0.36)	0.36	(0.36, 0.37)	0.03	(0.03, 0.03)	8	(8, 9)
Stance	CHF	1.34	(1.31, 1.38)	1.34	(1.31, 1.38)	0.02	(0.02, 0.02)	1	(1, 2)
	COPD	1.35	(1.33, 1.37)	1.35	(1.33, 1.37)	0.02	(0.02, 0.02)	2	(2, 2)
	MS	1.39	(1.38, 1.40)	1.39	(1.38, 1.40)	0.02	(0.02, 0.02)	1	(1, 1)
	PD	1.20	(1.19, 1.21)	1.20	(1.19, 1.21)	0.01	(0.01, 0.01)	1	(1, 1)
	PFF	1.24	(1.22, 1.25)	1.24	(1.22, 1.25)	0.02	(0.02, 0.02)	2	(2, 2)
	HA	1.20	(1.19, 1.21)	1.20	(1.19, 1.21)	0.02	(0.02, 0.02)	2	(1, 2)

Values represent the mean and 95% confidence interval of all stances, swings, and strides of the test subjects for the given cohort. CHF, congestive heart failure; COPD, chronic obstructive pulmonary disease; DL, deep learning; HA, healthy adults; MS, multiple sclerosis; PD, Parkinson's disease; PFF, proximal femoral fracture.

## 4. Discussion

The specific aim of the current study was to determine whether a previously in-lab validated DL-based gait event detection algorithm (34) could be used for the detection of gait events from real-life walking bouts in a heterogeneous cohort of different mobility-limiting diseases. For that purpose, participants from different disease cohorts (CHF, COPD, MS, PD, and PFF) and a cohort of healthy adults were equipped with the INDIP system that consisted of PIs and IMUs for both feet. Participants wore the INDIP system for 2.5 h in the habitual environment, as chosen by the participants, and a wide range of activities were recorded in these ecologically valid environments. Data from the PIs and IMUs were used to generate reference timings for ICs and FCs, whereas raw data from the accelerometer and gyroscope were used as the input to the DL algorithm to identify ICs and FCs.

The recall and precision of gait events were used as a general measure for the detection performance and were considered high (i.e., recall ≥ 98% and precision ≥ 96%). For comparison, in Trojaniello et al. (27), no missed or extra gait events were observed in a heterogeneous sample of elderly, hemiparetic, parkinsonian, and choreic gait, but data were only collected from walking back and forth for 1 min in a 12 m walkway. Similarly, high recall and precision (≥98%) were reported for a continuous wavelet transform (CWT)-based algorithm, but it was evaluated only for 13 healthy participants and 3 hemiplegic participants who walked continuously along a 10 m walkway (86). A recent study (45) found a recall of 96% and precision of 89% in a cohort of 28 PD participants, who wore two IMUs on the feet for 2 weeks, which are slightly lower than the recall and precision from the current study. Overall, the data of the studies presented here, including the present study, indicate that very high recall and precision values can be achieved with the deep learning approach for the detection of gait events. This, together with the higher flexibility of the DL-based algorithms compared to conventional algorithms, speaks for the future use of such algorithms for the detection of gait in mobility-limiting diseases also in the habitual environment.

For the correctly detected gait events, the time differences between the predicted event timing and the annotated event timings were quantified as a measure of temporal agreement between the reference system and the DL-based algorithm. The time differences were still in the same range as those previously reported for CWT-based (23, 27, 30, 86, 87) and DL-based algorithms (34) validated on in-lab gait data. To put this into perspective, studies that evaluated the time differences of detected gait events from PIs when compared to force plates or instrumented walkways also reported time differences in the range from 0.02 s to 0.04 s (17, 64, 87). For the INDIP pressure insole method, a negligible delay (< 10 ms) was observed for FCs, and a consistent IC anticipation (20 ms) was found when compared to force plates (64). It suggests that a certain margin of uncertainty should be considered when interpreting gait event timing differences in the DL-based algorithm.

Finally, stride-specific gait parameters were derived for the correctly detected events. These may be of greatest clinical relevance since changes in spatiotemporal gait parameters were associated with a shorter time to PD diagnosis (88) and from mild cognitive impairment to Alzheimer's disease (89), and values of temporal gait parameters were different in disease cohorts compared to healthy cohorts (90–92). Here, a zero-mean time difference was found for the stride times for all cohorts. Similarly, the time differences for stance and swing times were centered around a zero-mean difference for all cohorts, with only the mean differences for the stance and swing time of the PFF cohort being a bit larger (0.07 s and −0.07 s for the stance and swing time, respectively). The mean differences for stance and swing times in the PFF cohort may in part be explained by the altered gait pattern that is observed in this cohort (93, 94). Nonetheless, the time agreement for the stride-specific temporal gait parameters derived from the DL algorithm and the reference system was in a similar range as those communicated before for a comparable DL-based approach that evaluated results only from straight-line walking in a supervised laboratory setting (55).

The very good results that were obtained in the current study for two-feet-worn IMUs (56) combined with the results for a single shank-worn IMU from our previous study (34) provided evidence that the algorithm performance generalizes to other sensor wear locations and to free-living gait data. The current algorithm has the additional benefit that it does not require the knowledge of exact sensor location and orientation relative to the feet contrary to many previously validated algorithms (23, 24, 31, 34, 95). This has the practical consequence that there are less stringent requirements for study participants or future patients on how to attach the sensors to their feet. Since for the previous validation the input data consisted of the raw accelerometer and gyroscope signals from a single sensor that was located either laterally above the ankle joint or medially below the knee joint (34), the algorithm for the current validation was again trained, validated, and tested. Both studies show a high recall and sensitivity, highlighting that the algorithm is capable of detecting gait events from different sensor locations without the loss of accuracy provided that sufficient training data are available for any new sensor location (34). Furthermore, the algorithm performance was evaluated across a broad spectrum of five different mobility-limiting disease cohorts, and although the number of participants in the testing set for each cohort was low, it showed that the algorithm was able to accurately detect gait events in heterogeneous pathological gait patterns. This will ultimately allow future users of the algorithm to perform not only sensitivity analyses for individual cohorts but also specificity analyses across different cohorts.

The limitation of the current study included that only data from detected WBs were used. This means that gait event detection relied on the accurate detection of gait sequences as a preceding step (45). However, several algorithms have been reported for accurate IMU-based gait sequence detection in both healthy and disease cohorts (24, 25, 28, 29, 46, 96–100). Furthermore, data from some participants had to be excluded from analysis due to missing or incomplete data which was mainly due to issues with the PIs. As reference timings for gait events are still obtained mainly from force or pressure measuring device (23), it showed the difficulty of obtaining a dataset with annotated gait events on completely unsupervised free-living gait data (35–37, 45). To get a better picture of the algorithm's generalizability to other datasets, it needs to be tested on newly unseen datasets, for example, with a slightly different sensor setup, such as in Martindale et al. (46).

In addition, the study did not evaluate clinical aspects in detail, such as medication and symptom fluctuations. This is, in part, due to the heterogeneous sample of participants with different mobility-limiting diseases. Consequently, the current study did not focus on identifying, for example, digital biomarkers of disease progression, for which a greater sample size of a specific disease would be required. However, as this is a study comparing, in the same person, systems at one point in time on a motility aspect, we believe that this does not influence the results reported here. Furthermore, it should be stressed that the heterogeneous sample is an asset of the current study as the results show that the algorithm achieves excellent performance for different pathological gait patterns. Given the time span of 2.5 h, we did not specifically investigate whether disease-associated gait abnormalities, such as freezing of gait in PD (101), were captured by the recording. However, the duration of the assessment was chosen as a trade-off between experimental, clinical, and technical requirements (56) and is five times longer than the recommendations for validation procedures of assessing physical activity in older adults (102). Lastly, the current analysis also relied on a peak detection algorithm to identify the most probable timings of gait events (34, 46, 55). However, from a clinical perspective, this may be regarded as a benefit since it would allow a clinician to decide whether to consider certain strides based on how confidently it can be assumed that it was indeed a stride.

## 5. Conclusion

This study aimed to validate a DL algorithm for the detection of gait events in an ecologically valid environment across different mobility-limiting disease cohorts. The performance evaluation showed an excellent detection rate and low time errors for both event timings and subsequently derived temporal gait parameters for all cohorts. The DL reached a performance that was in a similar range or slightly better than approaches that were to date only validated on in-lab recorded gait data or for a specific disease cohort.

As the DL algorithm does not rely on expert-defined decision rules or hand-crafted features nor on exact sensor-to-segment alignment, it poses fewer requirements on the data collection.

Our next steps include extending the current analysis for data from multiple days and evaluating to which extent the DL network can be trained using participant-specific data to improve gait event detection on an individual level. Future studies may also consider the development of novel gold-standard systems that allow validation approaches beyond lower limb movement, for example, to include upper limb movement.

## Data availability statement

The data analyzed in this study is subject to the following licenses/restrictions: The Mobilise-D consortium are planning to make representative data available through online repositories (Zenodo & GitHub) in the near future. All scripts that were designed for the current analysis are available online and can be found at: GitHub, https://github.com/neurogeriatricskiel/mobilised. Requests to access these datasets should be directed to RR, r.romijnders@neurologie.uni-kiel.de.

## Ethics statement

The studies involving humans were approved by (1) Ethical Committee of the Medical Faculty of Kiel University, D438/18; (2) Helsinki Committee, Tel Aviv Sourasky Medical Center, Tel Aviv, Israel, 0551-392 19TLV; (3) Ethical Committee of the Medical Faculty of The University of Tuebingen, 647/2019BO2; and (4) London—Bloomsbury Research Ethics Committee, 19/LO/1507. The studies were conducted in accordance with the local legislation and institutional requirements. Written informed consent for participation was not required from the participants or the participants' legal guardians/next of kin in accordance with the national legislation and institutional requirements.

## Author contributions

RR, FS, CH, ACe, GS, and WM: conceptualization and project administration. RR, FS, ACe, and GS: methodology. RR and FS: software. RR: validation, formal analysis, writing—original draft preparation, visualization, and investigation. ACe and WM: resources and funding acquisition. RR, FS, and ACe: data curation. FS, CH, ACe, GS, and WM: writing—reviewing and editing. GS and WM: supervision. RR, FS, CH, AKü, AP-I, ACe, LA, KA, CB, SB, TB, PB, EB, ACa, A-EC, MC, BC, LC, ID'A, SD, BE, SF, MF, JG, EG, JH, HH, EH, AKe, CK, FK, SK, CM, DM, EM-A, AM, LP, LR, LS, KS, BS, DS, AS, MU, BV, IV, AY, GS, and WM: intellectual contribution. All authors have read and agreed to the published version of the manuscript.
